# Enhanced LogTODIM-TOPSIS framework for interval-valued intuitionistic fuzzy MAGDM and applications to intangible assets operational management performance evaluation of commercial sporting events

**DOI:** 10.1016/j.heliyon.2024.e26311

**Published:** 2024-02-16

**Authors:** Ke Xu, Kyunghwan Choi, Fengshuo Rao

**Affiliations:** a General Graduate School, Dongshin University, Naju, 58245, Jeollanam-do province, Republic of Korea; b Department of Sports and Leisure, Dongshin University, Naju, 58245, Jeollanam-do Province, Republic of Korea

**Keywords:** MAGDM issue, IVIFSs, LogTODIM technique, TOPSIS technique, Performance evaluation

## Abstract

The effective operation of intangible assets in commercial sports events can bring long-term, sustainable, and large-scale economic benefits to sports events. However, currently, in the operation of commercial sports events, the lack of experience in hosting events often hinders the development of intangible assets that should have created higher profits, and cannot achieve the expected value. The intangible assets operational management performance (IAOMP) evaluation of commercial sporting events is the multiple-attribute group decision-making (MAGDM). The Logarithmic TODIM (LogTODIM) and TOPSIS technique was brought forward the MAGDM. The interval-valued intuitionistic fuzzy sets (IVIFSs) are brought forward as the useful technique for coping with uncertain and fuzzy information during the IAOMP evaluation of commercial sporting events. In this study, the interval-valued intuitionistic fuzzy number Logarithmic TODIM-TOPSIS (IVIFN-LogTODIM-TOPSIS) technique is brought forward the MAGDM under IVIFSs circumstances. Finally, the numerical example for IAOMP evaluation of commercial sporting events is brought forward to verify the IVIFN-LogTODIM-TOPSIS technique. The main contribution of this study is brought forward: (1) the LogTODIM-TOPSIS was extended to IVIFSs in light with MEREC model; (2) the MEREC model is brought forward to derive weight in light with score information values under IVIFSs circumstances. (3) the IVIFN-LogTODIM-TOPSIS is brought forward for MAGDM under IVIFSs circumstances; (4) the numerical example for IAOMP evaluation of commercial sporting events and several different comparative analysis is brought forward to verify the IVIFN-LogTODIM-TOPSIS model.

## 1 Introduction

At present, the managers of some commercial sports event organizers attach great importance to the physical assets of the event, but their understanding of the importance of intangible assets of the event is not sufficient [[1], [2], [3]]. At the same time, even managers of commercial sports events are aware of the value of intangible assets, but they are not very familiar with the scope of intangible assets. They often only consider the event logo and broadcasting rights as intangible assets, while ignoring intangible assets with huge development potential and appreciation space such as event image, content flow, marketing network, and human resources [[4], [5], [6]]. This leads to a lack of systematic and holistic development planning for commercial sports events, as well as a lack of ability to create intangible assets, resulting in economic losses for the organizers. Taking the products provided by clubs in the CBA basketball league as an example, many clubs in the CBA basketball league do not have a true understanding of consumer needs, cannot recognize the important value of intangible assets in the competition, and have not been able to promote them in a targeted manner, resulting in a low conversion rate of intangible asset value in the competition. In the process of operating intangible assets in commercial sports events, legal support and protection are indispensable [[7], [8], [9]]. However, due to issues such as weak legal awareness and limited understanding of intangible asset categories among commercial sports event organizers, the risk of legal disputes remains latent, which is highly likely to cause economic and social losses to the organizers and their partners [[10], [11], [12]]. With the increasing importance of intangible assets management in sports events, legal disputes related to the operation of intangible assets management in commercial sports events are also increasing. This highlights the importance of enhancing the protection awareness of intangible assets management in sports events [[13], [14], [15]]. As the organizers of commercial sports events, they should continuously enhance their legal awareness during the operation of intangible assets management in sports events, in order to create and protect intangible assets management in sports events. If the host misses the opportunity to protect the intangible assets management of the event, it is highly likely to face the situation of counterfeit events, and their own image will be damaged due to improper behavior of counterfeit events [[16], [17], [18]]. The operation of intangible assets management in commercial sports events by the host is the only way to enhance the soft power of the event and obtain economic benefits. The operation of intangible assets management in commercial sports events is mainly constrained by four factors: the creative ability, protection awareness, management awareness, and operation method of the event's intangible assets management by the host. In order to effectively enhance the operational benefits of intangible assets management in commercial sports events, targeted creation of intellectual intangible assets management in sports events can be achieved through segmented target markets in practice; Strengthen the operation of human and management intangible assets management in competitions; Select partners and utilize intangible assets of the competition to achieve cross-border development [[19], [20], [21]].

Decision making is a fundamental activity in today's human society [[22], [23], [24], [25]]. In a highly democratic and free social environment, decisions can be reflected in various fields such as purchasing valuable items for families, selecting investment projects for companies, selecting locations for express delivery centers, selecting design solutions, and making national development strategy decisions [[26], [27], [28], [29], [30]]. A good decision can lead to good development for a family, business, or country. On the contrary, a bad decision can have a negative impact on the development of things, and even cause extremely bad consequences [[31], [32], [33], [34], [35]]. With the continuous development of social economy and technology, the decision-making environment is becoming increasingly complex, and the evaluation criteria for decision-making problems are becoming more diverse [[36], [37], [38], [39], [40]]. Various criteria are mutually restrictive or even contradictory. Relying on decision-makers to make decisions through a single evaluation criterion is meaningless, which has led to more and more scholars dedicating themselves to the study of MCDM [[41], [42], [43]]. MCDM problems can be divided into two categories: multi-objective decision-making (MODM) problems and MADM problems [[44], [45], [46]]. Among them, the decision samples for the MODM problem are continuous, with the goal of designing the optimal solution [[47], [48], [49], [50], [51], [52]]. The decision samples for MADM problems are discrete, with the aim of selecting from a limited number of alternative solutions to obtain the optimal solution Obviously, the latter is more common in practical decision-making problems [[53], [54], [55]]. However, as the reality turns out to be complex, it is not easy for the single-expert to manage the effectiveness of MADM. Therefore, MAGDM with multiple-people participating in MADM has emerged both domestically and internationally [[56], [57], [58], [59], [60]]. The IAOMP evaluation of commercial sporting events is adapted as the MAGDM issue. The IVIFSs [61] are brought forward as the technique for coping with uncertain and fuzzy information during IAOMP evaluation of commercial sporting events. Furthermore, several existing techniques brought forward the traditional LogTODIM [62] and TOPSIS technique [63] to implement the MADM. Until now, no or few research techniques were brought forward on MEREC model [64] and LogTODIM-TOPSIS technique under IVIFSs circumstances. Therefore, the combined IVIFN-LogTODIM-TOPSIS technique is brought forward the MAGDM under IVIFSs circumstances. The numerical example for IAOMP evaluation of commercial sporting events and several different comparative analysis is brought forward to verify the IVIFN-LogTODIM-TOPSIS. The main aim and motivation of this study is brought forward: (1) the LogTODIM-TOPSIS technique was expanded to IVIFSs in light with MEREC model; (2) the MEREC model is brought forward to derive weight in light with score information values under IVIFSs circumstances. (3) the IVIFN-LogTODIM-TOPSIS is brought forward MAGDM under IVIFSs circumstances; (4) a numerical example for IAOMP evaluation of commercial sporting events and several different comparative analysis is brought forward to verify the IVIFN-LogTODIM-TOPSIS.

The framework of this study is adapted. In Sect. 2, the IVIFSs is adapted. In Sect. 3, IVIFN-LogTODIM-TOPSIS is brought forward under IVIFSs circumstances. Sect. 4 brought forward the numerical example for IAOMP evaluation of commercial sporting events and several different comparative analysis. Some research remarks are adapted in Sect. 5.

## 2 Preliminaries

Atanassov [61] adapted the IVIFSs.

Definition 1 [61]. The IVIFSs is adapted in Eq. (1):

(1) WI={⟨θ,WM(θ),WN(θ)⟩|θ∈Θ}

where WM(θ)⊂[0,1] is adapted as membership degree and WN(θ)⊂[0,1] is adapted as non-membership degree, 0≤supWM(θ)+supWN(θ)≤1, ∀θ∈Θ. For convenience, WI=([WA,WB],[WC,WD]) is adapted as the IVIFN.

Definition 2 [65]. Let $WI_{1}=\left(\left[WA_{1},WB_{1}\right],\left[WC_{1},WD_{1}\right]\right)$ and $WI_{2}=\left(\left[WA_{2},WB_{2}\right],\left[WC_{2},WD_{2}\right]\right)$ be IVIFNs, the operation laws are adapted in Eq. (2)- Eq. (5):

(2) $$WI_{1}⨁WI_{2}=\left(\begin{array}{l} \left[WA_{1}+WA_{2}-WA_{1}WA_{2},WB_{1}+WB_{2}-WB_{1}WB_{2}\right],\\ \left[WC_{1}WC_{2},WD_{1}WD_{2}\right] \end{array}\right)$$

(3) $$WI_{1}⨂WI_{2}=\left(\begin{array}{l} \left[WA_{1}WA_{2},WB_{1}WB_{2}\right],\\ \left[WC_{1}+WC_{2}-WC_{1}WC_{2},WD_{1}+WD_{2}-WD_{1}WD_{2}\right] \end{array}\right)$$

(4) $$\xi WI_{1}=\left(\left[1-{\left(1-WA_{1}\right)}^{\xi },1-{\left(1-WB_{1}\right)}^{\xi }\right],\left[{\left(WC_{1}\right)}^{\xi },{\left(WD_{1}\right)}^{\xi }\right]\right),\xi >0$$

(5) $$WI_{1}^{\xi }=\left(\left[{\left(WA_{1}\right)}^{\xi },{\left(WB_{1}\right)}^{\xi }\right],\left[1-{\left(1-WC_{1}\right)}^{\xi },1-{\left(1-WD_{1}\right)}^{\xi }\right]\right),\xi >0$$

In line with Definition 2, several properties are adapted.

$$\left(1\right)\;WI_{1}⊕WI_{2}=WI_{2}⊕WI_{1},WI_{1}⊗WI_{2}=WI_{2}⊗WI_{1},{\left({\left(WI_{1}\right)}^{\xi_{1}}\right)}^{\xi_{2}}={\left(WI_{1}\right)}^{\xi_{1}\xi_{2}}\text{;}$$

$$\left(2\right)\;\xi \left(WI_{1}⊕WI_{2}\right)=\xi WI_{1}⊕\xi WI_{2},{\left(WI_{1}⊗WI_{2}\right)}^{\xi }={\left(WI_{1}\right)}^{\xi }⊗{\left(WI_{2}\right)}^{\xi }\text{;}$$

$$\left(3\right)\;\xi_{1}WI_{1}⊕\xi_{2}WI_{1}=\left(\xi_{1}+\xi_{2}\right)WI_{1},{\left(WI_{1}\right)}^{\xi_{1}}⊗{\left(WI_{1}\right)}^{\xi_{2}}={\left(WI_{1}\right)}^{\left(\xi_{1}+\xi_{2}\right)}\text{.}$$

Definition 3 [66]. Let $WI_{1}=\left(\left[WA_{1},WB_{1}\right],\left[WC_{1},WD_{1}\right]\right)$ and $WI_{2}=\left(\left[WA_{2},WB_{2}\right],\left[WC_{2},WD_{2}\right]\right)$ be designed IVIFNs, the score information values (SIV) and accuracy information values (AIV) of $WI_{1}$ and $WI_{2}$ are adapted Eq. (6)- Eq. (9):

(6) $$SIV\left(WI_{1}\right)=\frac{WA_{1}+WA_{1}\left(1-WA_{1}-WC_{1}\right)+WB_{1}+WB_{1}\left(1-WB_{1}-WD_{1}\right)}{2}$$

(7) $$SIV\left(WI_{2}\right)=\frac{WA_{2}+WA_{2}\left(1-WA_{2}-WC_{2}\right)+WB_{2}+WB_{2}\left(1-WB_{2}-WD_{2}\right)}{2}$$

(8) $$AIV\left(WI_{1}\right)=\frac{WA_{1}+WC_{1}+WB_{1}+WD_{1}}{2}\text{,}$$

(9) $$AIV\left(WI_{2}\right)=\frac{WA_{2}+WC_{2}+WB_{2}+WD_{2}}{2}$$

For $WI_{1}$ and $WI_{2}$, in line with Definition 3, then

$$\begin{array}{l} \left(1\right)\;if\;SIV\left(WI_{1}\right)<SIV\left(WI_{2}\right),WI_{1}<WI_{2}\text{;}\\ \left(2\right)\;if\;SIV\left(WI_{1}\right)>SIV\left(WI_{2}\right),WI_{1}>WI_{2}\text{;}\\ \left(3\right)\;if\;SIV\left(WI_{1}\right)=SIV\left(WI_{2}\right),AIV\left(WI_{1}\right)<AIV\left(WI_{2}\right),WI_{1}<WI_{2}\text{;}\\ \left(4\right)\;if\;SIV\left(WI_{1}\right)=SIV\left(WI_{2}\right),AIV\left(WI_{1}\right)=AIV\left(WI_{2}\right),WI_{1}=WI_{2}\text{.} \end{array}$$

Definition 4 [67]. Let $WI_{1}=\left(\left[WA_{1},WB_{1}\right],\left[WC_{1},WD_{1}\right]\right)$ and $WI_{2}=\left(\left[WA_{2},WB_{2}\right],\left[WC_{2},WD_{2}\right]\right)$, the IVIFN combined distance (IVIFNCD) is adapted Eq. (10):

(10) $$IVIFNCD\left(WI_{1},WI_{2}\right)=\frac{1}{2}\left(\begin{array}{l} \sqrt{\frac{1}{4}\left[\begin{array}{l} {\left(WA_{1}-WA_{2}\right)}^{2}+{\left(WB_{1}-WB_{2}\right)}^{2}\\ +{\left(WC_{1}-WC_{2}\right)}^{2}+{\left(WD_{1}-WD_{2}\right)}^{2} \end{array}\right]}\\ +\frac{1}{4}\left[\begin{array}{l} \left|WA_{1}-WA_{2}\right|+\left|WB_{1}-WB_{2}\right|\\ +\left|WC_{1}-WC_{2}\right|+\left|WD_{1}-WD_{2}\right| \end{array}\right] \end{array}\right)$$

The IVIFWA and IVIFWG model is adapted [68].

Definition 5 [68]. Let $WI_{j}=\left(\left[WA_{j},WB_{j}\right],\left[WC_{j},WD_{j}\right]\right)\left(j=1,2,3,\cdots ,n\right)$ be IVIFNs, the IVIFWA model is adapted Eq. (11):

(11) $$IVIFWA_{wa}\left(WI_{1},WI_{2},\ldots ,WI_{n}\right)=\overset{n}{\underset{j=1}{⊕}}\left(wa_{j}WI_{j}\right)=\left(\left[1-\prod_{j=1}^{n}{\left(1-WA_{j}\right)}^{wa_{j}},1-\prod_{j=1}^{n}{\left(1-WB_{j}\right)}^{wa_{j}}\right],\left[\prod_{j=1}^{n}{\left(WC_{j}\right)}^{wa_{j}},\prod_{j=1}^{n}{\left(WD_{j}\right)}^{wa_{j}}\right]\right)$$

Definition 6 where $wa={\left(wa_{1},wa_{2},...,wa_{n}\right)}^{T}$ be weight values of $WI_{j}=\left(\left[WA_{j},WB_{j}\right],\left[WC_{j},WD_{j}\right]\right)$, $wa_{j}>0,\sum_{j=1}^{n}wa_{j}$[69]. Let $WI_{j}=\left(\left[WA_{j},WB_{j}\right],\left[WC_{j},WD_{j}\right]\right)\left(j=1,2,3,\cdots ,n\right)$ be IVIFNs, the IVIFWG model is adapted Eq. (12):

(12) $$IVIFWG_{wa}\left(WI_{1},WI_{2},\ldots ,WI_{n}\right)=\overset{n}{\underset{j=1}{⊗}}{\left(WI_{j}\right)}^{wa_{j}}=\left(\begin{array}{l} \left[\prod_{j=1}^{n}{\left(WA_{j}\right)}^{wa_{j}},\prod_{j=1}^{n}{\left(WB_{j}\right)}^{wa_{j}}\right],\\ \left[1-\prod_{j=1}^{n}{\left(1-WC_{j}\right)}^{wa_{j}},1-\prod_{j=1}^{n}{\left(1-WD_{j}\right)}^{wa_{j}}\right] \end{array}\right)$$

where $wa={\left(wa_{1},wa_{2},...,wa_{n}\right)}^{T}$ be weight values of $WI_{j}=\left(\left[WA_{j},WB_{j}\right],\left[WC_{j},WD_{j}\right]\right)$, $wa_{j}>0,\sum_{j=1}^{n}wa_{j}$3IVIFN-LogTODIM-TOPSIS technique for MAGDM with MEREC

### 3.1 IVIF-MAGDM issues

**Fig. 1:**
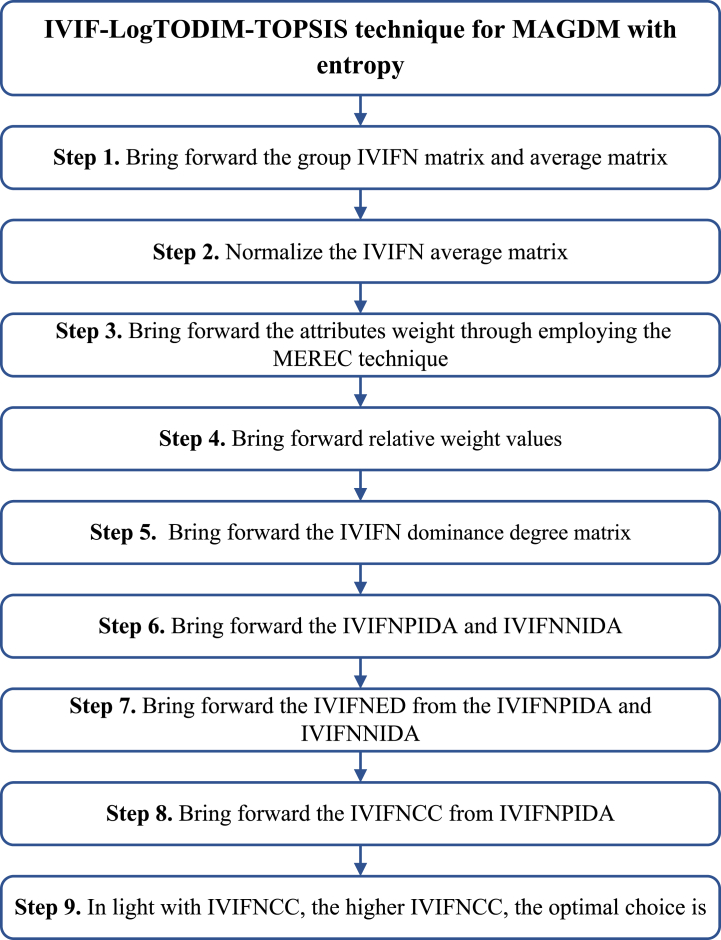
IVIFN-LogTODIM-TOPSIS technique for MAGDM with MEREC.

The IVIFN-LogTODIM-TOPSIS model is brought forward MAGDM. Let $WA=\left\{WA_{1},WA_{2},\cdots ,WA_{m}\right\}$ be alternatives and $WG=\left\{WG_{1},WG_{2},\cdots ,WG_{n}\right\}$ the attributes with weight $wa=\left(wa_{1},wa_{2},\cdots ,wa_{n}\right)$, $wa_{j}\in \left[0,1\right]$, $\sum_{j=1}^{n}wa_{j}=1$ and invited experts $WE=\left\{WE_{1},WE_{2},\cdots ,WE_{q}\right\}$ with weight $\omega a=\left(\omega a_{1},\omega a_{2},\cdots ,\omega a_{q}\right)$, $\omega a_{k}\in \left[0,1\right]$, $\sum_{k=1}^{q}\omega a_{q}=1$. Then, IVIFN-LogTODIM-TOPSIS technique is brought forward for MAGDM issue (See Fig. 1).

Step 1 Bring forward the IVIF-matrix $WR^{\left(t\right)}={\left[WR_{ij}^{\left(t\right)}\right]}_{m\times n}={\left(\left[WA_{ij}^{\left(t\right)},WB_{ij}^{\left(t\right)}\right],\left[WC_{ij}^{\left(t\right)},WD_{ij}^{\left(t\right)}\right]\right)}_{m\times n}$ and bring forward the average matrix $WR={\left[WR_{ij}\right]}_{m\times n}={\left(\left[WA_{ij},WB_{ij}\right],\left[WC_{ij},WD_{ij}\right]\right)}_{m\times n}$ in Eq. (13)- Eq. (14):

(13) $$\begin{array}{l} \begin{array}{cccc} \;WG_{1} & \;WG_{2} & \ldots & WG_{n} \end{array}\\ WR^{\left(t\right)}={\left[WR_{ij}^{\left(t\right)}\right]}_{m\times n}=\begin{array}{c} WA_{1}\\ WA_{2}\\ \vdots \\ WA_{m} \end{array}\left[\begin{array}{cccc} WR_{11}^{\left(t\right)} & WR_{12}^{\left(t\right)} & \ldots & WR_{1n}^{\left(t\right)}\\ WR_{21}^{\left(t\right)} & WR_{22}^{\left(t\right)} & \ldots & WR_{2n}^{\left(t\right)}\\ \vdots & \vdots & \vdots & \vdots \\ WR_{m1}^{\left(t\right)} & WR_{m2}^{\left(t\right)} & \ldots & WR_{mn}^{\left(t\right)} \end{array}\right] \end{array}$$

(14) $$\begin{array}{l} \begin{array}{cccc} \;WG_{1} & \;WG_{2} & \ldots & WG_{n} \end{array}\\ WR={\left[WR_{ij}\right]}_{m\times n}=\begin{array}{c} WA_{1}\\ WA_{2}\\ \vdots \\ WA_{m} \end{array}\left[\begin{array}{cccc} WR_{11} & WR_{12} & \ldots & WR_{1n}\\ WR_{21} & WR_{22} & \ldots & WR_{2n}\\ \vdots & \vdots & \vdots & \vdots \\ WR_{m1} & WR_{m2} & \ldots & WR_{mn} \end{array}\right] \end{array}$$

In light with IVIFWG technique, the $WR={\left[WR_{ij}\right]}_{m\times n}={\left(\left[WA_{ij},WB_{ij}\right],\left[WC_{ij},WD_{ij}\right]\right)}_{m\times n}$ is brought forward in Eq. (15):

(15) $$WR_{ij}={\left(WR_{ij}^{\left(1\right)}\right)}^{\omega a_{1}}⊗{\left(WR_{ij}^{\left(2\right)}\right)}^{\omega a_{2}}⊗\cdots ⊗{\left(WR_{ij}^{\left(q\right)}\right)}^{\omega a_{q}}=\left(\begin{array}{l} \left[\prod_{k=1}^{q}{\left(WA_{ij}^{\left(k\right)}\right)}^{\omega a_{k}},\prod_{k=1}^{q}{\left(WB_{ij}^{\left(k\right)}\right)}^{\omega a_{k}}\right],\\ \left[1-\prod_{k=1}^{q}{\left(1-WC_{ij}^{\left(k\right)}\right)}^{\omega a_{k}},1-\prod_{k=1}^{q}{\left(1-WD_{ij}^{\left(k\right)}\right)}^{\omega a_{k}}\right] \end{array}\right)$$

Step 2 The $WR={\left[WR_{ij}\right]}_{m\times n}={\left(\left[WA_{ij},WB_{ij}\right],\left[WC_{ij},WD_{ij}\right]\right)}_{m\times n}$ is normalized to $NWR={\left[NWR_{ij}\right]}_{m\times n}={\left(\left[NWA_{ij},NWB_{ij}\right],\left[NWC_{ij},NWD_{ij}\right]\right)}_{m\times n}$ in Eq. (16)- Eq. (17):

For benefit attributes:

(16) $$NWR_{ij}=\left(\left[NWA_{ij},NWB_{ij}\right],\left[NWC_{ij},NWD_{ij}\right]\right)=\left(\left[WA_{ij},WB_{ij}\right],\left[WC_{ij},WD_{ij}\right]\right)$$

For cost attributes:

(17) $$NWR_{ij}=\left(\left[NWA_{ij},NWB_{ij}\right],\left[NWC_{ij},NWD_{ij}\right]\right)=\left(\left[WC_{ij},WD_{ij}\right],\left[WA_{ij},WB_{ij}\right]\right)$$

### 3.2 Bring forward the attributes weight through MEREC model

Step 3 Bring forward the attributes weight through employing the MEREC model [64].

(1) Bring forward the normalized IVIFN score values matrix (NIVIFNSVM) in Eq. (18):

(18) $$NIVIFNSVM\left(NWR_{ij}\right)=\frac{\min_{i}SIV\left(NWR_{ij}\right)}{SIV\left(NWR_{ij}\right)}$$

(2) Bring forward the overall information performance $NIVIFNSVM\left(NWR_{i}\right)$ in Eq. (19).

(19) $$NIVIFNSVM\left(NWR_{ij}\right)=\ln\left(1+\left(\frac{1}{n}\sum_{j=1}^{n}\left|\ln\;NIVIFNSVM\left(NWR_{ij}\right)\right|\right)\right),i=1,2,3,\cdots ,m\text{.}$$

(3) Bring forward the overall performance of $WA_{i}$ through removing each attribute in Eq. (20).

$$NIVIFNSVM\left(NWR_{i\left(j\right)}\right)=\ln\left(1+\left(\frac{1}{n}\sum_{k=1,k\neq j}^{n}\left|\ln\;NIVIFNSVM\left(NWR_{ik}\right)\right|\right)\right)$$

(20) i=1,2,3,⋯,m;k=1,2,3,⋯,n,

(4) Bring forward the sum of absolute deviation information in Eq. (21):

(21) $$IVIFNSD_{j}=\sum_{i=1}^{m}\left|NIVIFNSVM\left(NWR_{i\left(j\right)}\right)-NIVIFNSVM\left(NWR_{i}\right)\right|$$

(5) Bring forward the weight values in Eq. (22):

(22) $$wa_{j}=\frac{IVIFNSD_{j}}{\sum_{j=1}^{n}IVIFNSD_{j}},j=1,2,3,\cdots n\text{.}$$

### 3.3 IVIFN-LogTODIM-TOPSIS model for MAGDM

Then, the IVIFN-LogTODIM-TOPSIS model is brought forward MAGDM.

Step 4 Bring forward relative weight values of $WG_{j}$ in Eq. (23):

(23) $$rwa_{j}=wa_{j}/\max_{j}ws_{j}\text{,}$$

Step 5 Bring forward the dominance information degree $DID={\left(DID_{ij}\right)}_{m\times n}$.

(1) The dominance information degree $DID_{j}\left(WA_{i},WA_{t}\right)$ of $WA_{i}$ over $WA_{t}$ for $WG_{j}$ is brought forward in Eq. (24):

(24) $$DID_{j}\left(WA_{i},WA_{t}\right)=\left\{ \begin{array}{cc} \frac{rwa_{j}\times \log\left(1+10\rho IVIFCD\left(NWR_{ij},NWR_{tj}\right)\right)}{\sum_{j=1}^{n}rwa_{j}} & if\;SIV\left(NWR_{ij}\right)>SIV\left(NWR_{tj}\right)\\ 0 & if\;SIV\left(NWR_{ij}\right)=SIV\left(NWR_{tj}\right)\\ -\frac{rwa_{j}\times \lambda \;\log\left(1+10\rho IVIFCD\left(NWR_{ij},NWR_{tj}\right)\right)}{\sum_{j=1}^{n}rwa_{j}} & if\;ISIV\left(NWR_{ij}\right)<SIV\left(NWR_{tj}\right) \end{array}\right.$$

where λ∈[1,5] and $\rho \in N^{+}$ is adapted in line with agent's perception [70].

(2) The $DID_{j}\left(WA_{i}\right)$ under $WG_{j}$ is brought forward in Eq. (25):

(25) $$\begin{array}{l} DID_{j}\left(WA_{i}\right)={\left[DID_{j}\left(WA_{i},WA_{t}\right)\right]}_{m\times m}\\ \begin{array}{cccc} \;WA_{1} & \;WA_{2} & \cdots & \;WA_{m} \end{array}\\ =\begin{array}{c} WA_{1}\\ WA_{2}\\ \vdots \\ WA_{m} \end{array}\left[\begin{array}{cccc} 0 & DID_{j}\left(DA_{1},DA_{2}\right) & \cdots & DID_{j}\left(DA_{1},DA_{m}\right)\\ DID_{j}\left(DA_{2},DA_{1}\right) & 0 & \cdots & DID_{j}\left(DA_{2},DA_{m}\right)\\ \vdots & \vdots & \cdots & \vdots \\ DID_{j}\left(DA_{m},DA_{1}\right) & DID_{j}\left(DA_{m},DA_{2}\right) & \cdots & 0 \end{array}\right] \end{array}$$

(3) Bring forward the $DID_{j}\left(WA_{i}\right)$ for other alternatives under $WG_{j}$ in Eq. (26):

(26) $$DID_{j}\left(WA_{i}\right)=\sum_{t=1}^{m}DID_{j}\left(WA_{i},WA_{t}\right)$$

The $DID={\left(DID_{ij}\right)}_{m\times n}$ is brought forward in Eq. (27):

(27) $$DID={\left(DID_{ij}\right)}_{m\times n}=\left[\begin{array}{ccccc} & WG_{1} & WG_{2} & \ldots & WG_{n}\\ WA_{1} & \sum_{t=1}^{m}DID_{1}\left(WA_{1},WA_{t}\right) & \sum_{t=1}^{m}DID_{2}\left(WA_{1},WA_{t}\right) & \ldots & \sum_{t=1}^{m}DID_{n}\left(WA_{1},WA_{t}\right)\\ WA_{2} & \sum_{t=1}^{m}DID_{1}\left(WA_{2},WA_{t}\right) & \sum_{t=1}^{m}DID_{2}\left(WA_{2},WA_{t}\right) & \ldots & \sum_{t=1}^{m}DID_{n}\left(WA_{2},WA_{t}\right)\\ \vdots & \vdots & \vdots & \vdots & \vdots \\ WA_{m} & \sum_{t=1}^{m}DID_{1}\left(WA_{m},WA_{t}\right) & \sum_{t=1}^{m}DID_{2}\left(WA_{m},WA_{t}\right) & \ldots & \sum_{t=1}^{m}DID_{n}\left(WA_{m},WA_{t}\right) \end{array}\right]$$

Step 6 Bring forward IVIFN positive ideal decision alternative (IVIFNPIDA) and IVIFN negative ideal decision alternative (IVIFNNIDA) in Eq. (28)- Eq. (30):

(28) $$IVIFNPIDA=\left(IVIFNPIDA_{1},IVIFNPIDA_{1},\cdots ,IVIFNPIDA_{n}\right)$$

(29) $$IVIFNNIDA=\left(IVIFNNIDA_{1},IVIFNNIDA_{1},\cdots ,IVIFNNIDA_{n}\right)$$

(30) $$IVIFNPIDA_{j}=\max_{j=1}^{n}DID_{ij},IVIFNNIDA_{j}=\min_{j=1}^{n}DID_{ij}$$

Step 7 Bring forward the IVIFN Euclidean distance (IVIFNED) from the IVIFNPIDA and IVIFNNIDA in Eq. (31)- Eq. (32):

(31) $$IVIFNED\left(WA_{i},IVIFNPIDA\right)=\sqrt{\sum_{j=1}^{n}{\left(DID_{ij}-IVIFNPIDA_{j}\right)}^{2}}$$

(32) $$IVIFNED\left(WA_{i},IVIFNNIDA\right)=\sqrt{\sum_{j=1}^{n}{\left(DID_{ij}-IVIFNNIDA_{j}\right)}^{2}}$$

Step 8 Bring forward the IVIFN closeness coefficient (IVIFNCC) from IVIFNPIDA in Eq. (33).

$$IVIFNCC\left(WA_{i},IVIFNPIDA\right)=\frac{IVIFNED\left(WA_{i},IVIFNNIDA\right)}{IVIFNED\left(WA_{i},IVIFNPIDA\right)+IVIFNED\left(WA_{i},IVIFNPIDA\right)}$$

(33) $$=\frac{\sqrt{\sum_{j=1}^{n}{\left(DID_{ij}-IVIFNNIDA_{j}\right)}^{2}}}{\sqrt{\sum_{j=1}^{n}{\left(DID_{ij}-IVIFNNIDA_{j}\right)}^{2}}+\sqrt{\sum_{j=1}^{n}{\left(DID_{ij}-IVIFNPIDA_{j}\right)}^{2}}}$$

Step 9 In light with IVIFNCC, the larger IVIFNCC, the optimal alternative is.

## 4 Numerical example and comparative analysis

### 4.1 Numerical example

As an important component of sports events, the sustainable development of commercial sports events is closely related to the overall output value of sports events. The branding development of commercial sports events requires the effective operation of intangible assets management in the event through the creation of event logos and image shaping by the event organizers. However, in recent years, due to the lack of experience and lack of understanding of the operational methods of commercial sports events in the actual operation of intangible assets management by event organizers, the value of intangible assets in sports events has not been fully realized. This study applies brand positioning theory and brand extension theory to propose strategic suggestions for the constraints on the use of intangible assets in commercial sports events, in order to better promote the improvement of market competitiveness in commercial sports events. Intangible assets of commercial sports events refer to non-physical assets owned or controlled by the organizers, which have uncertainty and timeliness and can bring sustained economic benefits. The main forms of expression include: sports event names, franchise rights for logo identification, advertising rights, broadcasting rights, media franchise rights, etc. According to the characteristics of intangible asset classification, commercial sports intangible assets management could be divided into four categories: intellectual, human, market, and management. The operation of intangible assets management in commercial sports events refers to the behavior of event organizers to maximize the value and social benefits of event intangible assets management through the creation, utilization, protection, and management of event intangible assets management, ultimately promoting the development of event branding. From the current situation of well-known sports event management, sponsorship is one of the important sources of event revenue. As a reward for the sponsoring enterprise, the advertising and naming rights of the event are generally obtained by the sponsor. Taking basketball matches as an example, advertising rights mainly include trademarks on players' chest and back clothing, which are mainly developed by various teams; Naming rights are mainly divided into event naming rights and team naming rights, with the former developed by the basketball association and the latter developed by various teams. Television broadcasting rights are the main source of income for major commercial sports events. Due to the long-term low level of development of broadcasting rights for basketball events in China, in the early stages of basketball professionalization, in order to promote the reform and development of basketball events in China, CCTV began broadcasting NBA games in 1986. The development of franchise rights for sports competitions, organizational titles, and logo marks is also an important channel for commercial sports events to obtain economic benefits. For example, in 1998, at the Asian Games, 30 companies purchased the franchise rights for the title and logo of the Chinese delegation. From this perspective, the main problems in the operation of intangible assets management in commercial sports events are relatively small development efforts, single channels, and insufficient funding. The development of intangible assets management by commercial sports event organizers mainly focuses on projects with high returns, such as naming rights, venue advertising rights, and advertising rights on player uniforms. There is still a lack of effective protection and management for intangible assets management with unclear or low returns. Therefore, the IAOMP evaluation of commercial sporting events is adapted to verify the IVIFN-LogTODIM-TOPSIS. Five potential table tennis sporting events $WA_{i}\left(i=1,2,3,4,5\right)$ to choose in light with four decision attributes [52,71]: ①WG_1_ is development speed of IAOM for table tennis sporting events; ②WG_2_ is cost expenditure of IAOM for table tennis sporting events; ③WG_3_ is social influence of IAOM for table tennis sporting events; ④WG_4_ is development efficiency of IAOM for table tennis sporting events sports events. The WG_2_ is cost attribute. The five potential table tennis sporting events $WA_{i}\left(i=1,2,3,4,5\right)$ are adapted in line with linguistic information scales (See Table 1 [72]) through three experts $WE_{t}\left(t=1,2,3\right)$ with weight values ωa=(0.3329,0.3507,0.3154).

**Table 1:** Linguistic information scales and IVIFNs.

Linguistic information scales	IVIFNs
Exceedingly Bad-WEB	⟨[0.0500,0.1000],[0.8500,0.9000]⟩
Very Bad -WVB	⟨[0.1000,0.1500],[0.7500,0.8500]⟩
Bad -WB	⟨[0.1500,0.2000],[0.6000,0.7000]⟩
Medium-WM	⟨[0.5000,0.5000],[0.5000,0.5000]⟩
Good-WG	⟨[0.6000,0.7000],[0.1500,0.2000]⟩
Very Good -WVG	⟨[0.7500,0.8500],[0.1000,0.1500]⟩
Exceedingly Good -WEG	⟨[0.8500,0.9000],[0.0500,0.1000]⟩

The IVIFN-LogTODIM-TOPSIS model is brought forward IAOMP evaluation of table tennis sporting events.

Step 1 Bring forward the IVIFN group matrix $WR={\left[WR_{ij}^{\left(k\right)}\right]}_{5\times 4}\left(k=1,2,3\right)$ (See Table 2, Table 3, Table 4).

**Table 2:** Linguistic scales through $WE_{1}$

	WG_1_	WG_2_	WG_3_	WG_4_
WA_1_	WVG	WVW	WB	WM
WA_2_	WVB	WW	WM	WG
WA_3_	WVG	WM	WB	WVB
WA_4_	WM	WVG	WVG	WVB
WA_5_	WM	WG	WVW	WVG

**Table 3:** Linguistic scales through $WE_{2}$

	WG_1_	WG_2_	WG_3_	WG_4_
WA_1_	WVG	WG	WB	WVB
WA_2_	WG	WM	WM	WB
WA_3_	WG	WM	WB	WVB
WA_4_	WM	WB	WVG	WVG
WA_5_	WVB	WB	WM	WG

**Table 4:** Linguistic scales through $WE_{3}$

	WG_1_	WG_2_	WG_3_	WG_4_
WA_1_	WVG	WM	WVB	WB
WA_2_	WVG	WVB	WM	WB
WA_3_	WVB	WB	WM	WG
WA_4_	WVB	WG	WVG	WM
WA_5_	WM	WVB	WG	WB

Then in light with the IVIFWG model, the $WR={\left[WR_{ij}\right]}_{5\times 4}$ is brought forward (See Table 5).

**Table 5:** The $WR={\left[WR_{ij}\right]}_{5\times 4}$

Alternatives	WG_1_	WG_2_
WA_1_	([0.5127,0.5487], [0.3216,0.4215])	([0.5142,0.5358], [0.3641,0.4621])
WA_2_	([0.3147,0.5946], [0.3698,0.3743])	([0.3178,0.5253], [0.3116,0.3519])
WA_3_	([0.3921,0.5884], [0.3142,0.4108])	([0.3973,0.5648], [0.3892,0.4312])
WA_5_	([0.3846,0.3132], [0.3987,0.5319])	([0.3498,0.5125], [0.3743,0.4606])
WA_5_	([0.3218,0.3657], [0.5419,0.6103])	([0.3591,0.5694], [0.3943,0.4313])
Alternatives	WG_3_	WG_5_
WA_1_	([0.2497,0.5157], [0.2937,0.3541])	([0.2324,0.5943], [0.2936,0.4012])
WA_2_	([0.5132,0.5498], [0.3652,0.4097])	([0.3435,0.5342], [0.4317,0.4606])
WA_3_	([0.2497,0.5316], [0.4102,0.4637])	([0.3476,0.5316], [0.2736,0.3963])
WA_5_	([0.3943,0.5875], [0.2937,0.4106])	([0.5713,0.5396], [0.2438,0.3645])
WA_5_	([0.5132,0.6253], [0.2887,0.3725])	([0.2649,0.3476], [0.2994,0.5348])

Step 2 Normalize the $WR={\left[WR_{ij}\right]}_{5\times 4}$ to $NWR={\left[NWR_{ij}\right]}_{5\times 4}$ (See Table 6).

**Table 6:** The $NWR={\left[NWR_{ij}\right]}_{5\times 4}$

Alternatives	WG_1_	WG_2_
WA_1_	([0.5127,0.5487], [0.3216,0.4215])	([0.3641,0.4621], [0.5142,0.5358])
WA_2_	([0.3147,0.5946], [0.3698,0.3743])	([0.3116,0.3519], [0.3178,0.5253])
WA_3_	([0.3921,0.5884], [0.3142,0.4108])	([0.3892,0.4312], [0.3973,0.5648])
WA_5_	([0.3846,0.3132], [0.3987,0.5319])	([0.3743,0.4606], [0.3498,0.5125])
WA_5_	([0.3218,0.3657], [0.5419,0.6103])	([0.3943,0.4313], [0.3591,0.5694])
Alternatives	WG_3_	WG_5_
WA_1_	([0.2497,0.5157], [0.2937,0.3541])	([0.2324,0.5943], [0.2936,0.4012])
WA_2_	([0.5132,0.5498], [0.3652,0.4097])	([0.3435,0.5342], [0.4317,0.4606])
WA_3_	([0.2497,0.5316], [0.4102,0.4637])	([0.3476,0.5316], [0.2736,0.3963])
WA_5_	([0.3943,0.5875], [0.2937,0.4106])	([0.5713,0.5396], [0.2438,0.3645])
WA_5_	([0.5132,0.6253], [0.2887,0.3725])	([0.2649,0.3476], [0.2994,0.5348])

Step 3 Bring forward the weight number: $wa_{1}=0.2909,wa_{2}=0.2903,wa_{3}=0.2602,wa_{4}=0.1559$.

Step 4 Bring forward relative weight: rwa=(0.9928,1.0000,0.8881,0.5321)

Step 5 Bring forward $DID={\left(DID_{ij}\right)}_{5\times 4}$ (See Table 7):

**Table 7:** The $DID={\left(DID_{ij}\right)}_{5\times 4}$

	WG_1_	WG_2_	WG_3_	WG_4_
WA_1_	0.8804	−1.6241	0.3491	−0.7581
WA_2_	−0.5336	1.2755	−1.9880	0.5680
WA_3_	−0.6350	−0.0861	−0.2452	1.0441
WA_4_	0.7288	−0.4969	0.2275	0.3463
WA_5_	−0.9846	−1.3965	−1.0846	−0.8812

Step 6 Bring forward IVIFNPIDA and IVIFNNIDA (See Table 8).

**Table 8:** The IVIFNPIDA and IVIFNNIDA.

	WG_1_	WG_2_	WG_3_	WG_4_
IVIFNPIDA	0.8804	1.2755	0.3491	1.0441
IVIFNNIDA	−0.9846	−1.6241	−1.9880	−0.8812

Step 7 Bring forward the $IVIFNED\left(WA_{i},IVIFNPIDA\right)$ and $IVIFNED\left(WA_{i},IVIFNNIDA\right)$ (See Table 9).

**Table 9:** The $IVIFNED\left(WA_{i},IVIFNPIDA\right)$ and $IVIFNED\left(WA_{i},IVIFNNIDA\right)$

Alternative	$IVIFNED\left(WA_{i},IVIFNPIDA\right)$	$IVIFNED\left(WA_{i},IVIFNNIDA\right)$
WA_1_	3.4140	2.9926
WA_2_	2.7728	3.2728
WA_3_	2.1222	3.0384
WA_4_	1.9147	3.2591
WA_5_	4.0473	0.9317

Step 8 Bring forward the $IVIFNCC\left(WA_{i},IVIFNPIDA\right)$ (See Table 10).

**Table 10:** The $IVIFNCC\left(WA_{i},IVIFNPIDA\right)$ and order.

Alternative	$IVIFNCC\left(WA_{i},IVIFNPIDA\right)$	Order
WA_1_	0.4671	4
WA_2_	0.5414	3
WA_3_	0.5888	2
WA_4_	0.6299	1
WA_5_	0.1871	5

Step 9 In light with the IVIFNCC, thus, the order is: $WA_{4}>WA_{3}>WA_{2}>WA_{1}>WA_{5}$ and the optimal table tennis sporting event is $WA_{4}$.

### 4.2 Comparative analysis

**Fig. 2:**
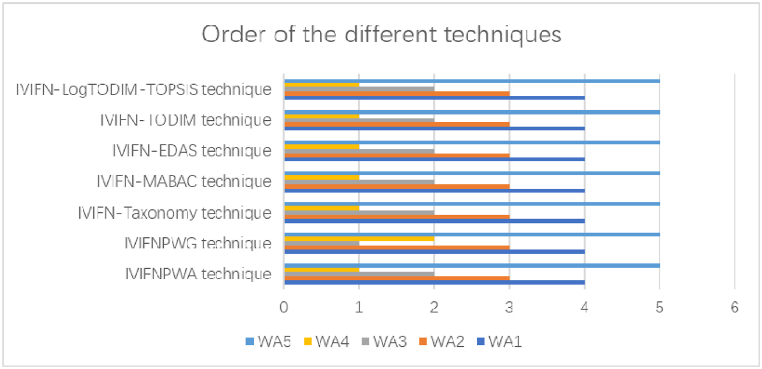
Order of different techniques.

Then, the IVIFN-LogTODIM-TOPSIS technique is compared with IVIFNPWA technique [73], IVIFNPWG technique [73], IVIFN-Taxonomy technique [74], IVIFN-MABAC technique [75], IVIFN-EDAS technique [76] and IVIFN-TODIM technique [77]. The comparative results are brought forward in Table 11 and Fig. 2.

**Table 11:** Order of different techniques.

Techniques	Order
IVIFNPWA technique [73]	$WA_{4}>WA_{3}>WA_{2}>WA_{1}>WA_{5}$
IVIFNPWG technique [73]	$WA_{4}>WA_{3}>WA_{1}>WA_{2}>WA_{5}$
IVIFN-Taxonomy technique [74]	$WA_{4}>WA_{3}>WA_{2}>WA_{1}>WA_{5}$
IVIFN-MABAC technique [75]	$WA_{4}>WA_{3}>WA_{2}>WA_{1}>WA_{5}$
IVIFN-EDAS technique [76]	$WA_{4}>WA_{3}>WA_{2}>WA_{1}>WA_{5}$
IVIFN-TODIM technique [77]	$WA_{4}>WA_{3}>WA_{2}>WA_{1}>WA_{5}$
IVIFN-LogTODIM-TOPSIS technique	$WA_{4}>WA_{3}>WA_{2}>WA_{1}>WA_{5}$

In light with RW coefficients [78](See Table 12), the RW coefficient information between IVIFNPWA technique [73], IVIFNPWG technique [73], IVIFN-Taxonomy technique [74], IVIFN-MABAC technique [75], IVIFN-EDAS technique [76], IVIFN-TODIM technique [77] and the IVIFN-LogTODIM-TOPSIS is 1.0000, 0.7917, 1.0000, 1.0000, 1.0000, 1.0000, respectively. This verifies the IVIFN-LogTODIM-TOPSIS technique is effective.

**Table 12:** WS coefficient calculation.

	IVIFN-LogTODIM-TOPSIS technique	IVIFNPWA technique	IVIFNPWG technique	IVIFN-Taxonomy technique
WA_1_	4	4	4	4
WA_2_	3	3	3	3
WA_3_	2	2	1	2
WA_4_	1	1	2	1
WA_5_	5	5	5	5
Coefficients	WS	1.0000	0.7917	1.0000
	IVIFN-LogTODIM-TOPSIS technique	IVIFN-MABAC technique	IVIFN-EDAS technique	IVIFN-TODIM technique
WA_1_	4	4	4	4
WA_2_	3	3	3	3
WA_3_	2	2	2	2
WA_4_	1	1	1	1
WA_5_	5	5	5	5
Coefficients	WS	1.0000	1.0000	1.0000

## 5 Conclusion

With the continuous promotion of the “Healthy China” strategy, “exercise is a good doctor” has gradually become a social consensus, and national fitness has become a major national strategy. According to the clear requirements of the “Healthy China 2030″ Plan Outline and the “Healthy China Action (2019–2030)", establishing an effective amateur competition activity system and incentive mechanism, and exploring a diversified mechanism for hosting competitions have become important measures to promote the progress of national fitness and accelerate the construction of a sports powerhouse. Against the backdrop of digital innovation and the flow of data elements, digital forms have become a key growth point for the transformation and upgrading of the sports industry, attracting attention from both capital and government in various fields such as fitness and leisure, education and training, sports media, and information services. The IAOMP evaluation in commercial sporting events is the MAGDM. The IVIFSs are brough forward useful technique for coping with uncertain and fuzzy information during IAOMP evaluation of commercial sporting events. In this study, the IVIFN-LogTODIM-TOPSIS model is brought forward MAGDM under IVIFSs circumstances. Finally, the numerical example for IAOMP evaluation of commercial sporting events is brought forward to verify the IVIFN-LogTODIM-TOPSIS model.

There may be some possible limitations of IAOMP evaluation of commercial sporting events, which may be explored in our future research contents: (1) It is a worthwhile research content to bring forward the prospect theory for IAOMP evaluation of commercial sporting events under IVIFSs circumstances [[79], [80], [81], [82]]; (2) It is also worthwhile research content to bring forward the regret theory for IAOMP evaluation of commercial sporting events under IVIFSs circumstances [[83], [84], [85], [86]]; (3) In subsequent research contents, the IAOMP evaluation of commercial sporting events needs to be brought forward in light with consensus measures [[87], [88], [89]].

## Ethics declaration statement

The authors state that this is their original work, and it is neither submitted nor under consideration in any other journal simultaneously.

## Data availability

The authors agree that the data used in this manuscript is available to everyone and anyone can use this data by just citing this article.

## Funding information

This study did not receive any funding in any form.

## CRediT authorship contribution statement

**Ke Xu:** Writing – original draft, Methodology, Data curation. **Kyunghwan Choi:** Supervision, Conceptualization. **Fengshuo Rao:** Writing – original draft, Methodology, Data curation.

## Declaration of competing interest

The authors declare that they have no known competing financial interests or personal relationships that could have appeared to influence the work reported in this paper.
